# Investigation of the Influence of Roughness and Dental Implant Design on Primary Stability via Analysis of Insertion Torque and Implant Stability Quotient: An In Vitro Study

**DOI:** 10.3390/jcm12134190

**Published:** 2023-06-21

**Authors:** Marta Romero, Mariano Herrero-Climent, Blanca Ríos-Carrasco, Aritza Brizuela, Manuel María Romero, Javier Gil

**Affiliations:** 1 Department of Periodontology, School of Dentistry, Universidad de Seville, C/Avicena S/N, 41009 Seville, Spain; romeromarta@uic.es (M.R.); dr.herrero@herrerocliment.com (M.H.-C.); brios@us.es (B.R.-C.); mmromero@infomed.es (M.M.R.); 2 Porto Dental Institute, Periodontology Department, Symmetrya Prothesis, Av. de Montevideu 810, 4150-518 Porto, Portugal; 3 Densia Reserach Group, Facultad de Ciencias de la Salud, Universidad Europea Miguel de Cervantes, C/del Padre Julio Chevalier 2, 47012 Valladolid, Spain; 4 Bioengineering Institute of Technology, Faculty of Medicine and Health Sciences, Universidad International de Cataluña, C/Josep Trueta s/n, Sant Cugat del Vallés, 08195 Barcelona, Spain

**Keywords:** dental implant, titanium, primary stability, insertion torque, implant stability quotient

## Abstract

In the placement of dental implants, the primary fixation between the dental implant and the bone is of great importance and corresponds to compressive mechanical fixation that aims to prevent micromovement of the implant. The aim of this research was to determine the role of roughness and the type of dental implant (tissue-level or bone-level) in implant stability, measured using resonance frequency analysis (RFA) and insertion torque (IT). We analyzed 234 titanium dental implants, placed in fresh calf ribs, at the half-tissue level and half-bone level. The implant surface was subjected to grit-blasting treatments with alumina particles of 120, 300, and 600 μm at a projection pressure of 2.5 bar, resulting in three types of roughness. Roughness was determined via optical interferometry. The wettability of the surfaces was also determined. Implant stability was measured using a high-precision torquemeter to obtain IT, and RFA was used to determine the implant stability quotient (ISQ). The results show that rough surfaces with Sa values of 0.5 to 4 μm do not affect the primary stability. However, the type of implant is important; bone-level implants obtained the highest primary stability values. A good correlation between the primary stability values obtained via IT and ISQ was demonstrated. New in vivo studies are necessary to know whether these results can be maintained in the long term.

## 1. Introduction

Several papers since the 1980s have confirmed the importance of the implant surface in implant osseointegration. Improved implant surface properties result in faster and stronger bone formation, which may confer better stability during the healing process. In this sense, a positive correlation has been found between surface roughness and bone-to-implant contact and pushout strength [1]. The final goal of implant surface modification is definitely to create a more osteophytic surface that attracts bone-forming cells [2].

This is the reason why researchers have tried to improve implant properties using methods such as roughening the implant surface using different techniques, such as subtractive and additive methods, including physical (turning and blasting), chemical (acid etching and alkalis), electrochemical (electropolishing and anodizing), deposition (plasma-spraying, sol–gel), and biochemical (proteins) methods [3,4]. Many of these studies have been performed with different dental implant macro-designs, lengths, diameters, thread depths, thread spacing, micro-threads, and other geometrical aspects of dental implants [5,6,7,8,9,10]. Conical or cylindrical implant shapes and the use of platform switching have also been studied, and in all cases, the authors determined the mechanical load transfer to the bone [11,12]. Different studies have reported a positive correlation between roughness and osseointegration, using machined surfaces as controls [13,14,15,16,17]. The roughness produces changes in the wettability, surface energy, zeta potential, and other physico-chemical properties that improve bone growth. [18,19,20,21,22].

Therefore, there is sufficient evidence of how implant surface modifications have achieved a stronger bone response, which may explain the high implant survival, and equally, many treatment indications have become more predictable, as in the case of the immediate placement and immediate loading of implants. Moreover, surface-modified implants also lead to the preservation of marginal bone, without the clinically significant superiority of any particular surface or design [23].

On the other hand, primary stability has been defined as the biometric stability measured immediately after implant insertion; it is determined by bone–implant contact, and is the result of the mechanical engagement of an implant with the surrounding bone. It is considered a prognostic marker for long-term implant success. Primary stability will be greater the smaller the micromovements between the surrounding bone and implant. This allows for proper and faster healing, and correct osseointegration [24].

Implant stability at the moment of implant insertion and the absence of micromovements are also critical to determining whether it is possible to immediately load the implant. Good fixation will prevent the formation of connective tissue between implant and bone. It is well known that certain micromovements cause the destruction of new cells and blood vessels. It has been suggested that micromotion between the implant and the surrounding bone must not exceed a threshold value of 150 μm for successful osseointegration. Any movement at the micrometer range above that threshold can induce stress and strain, hinder the recruitment of new cells, and negatively influence healing bone and bone remodeling, leading to the formation of fibrous tissue [24,25,26,27].

Different methods have been utilized to measure the stability of implants, among them, resonance frequency analysis (RFA), with the Osstell device (Osstell AB, Goteborg, Sweden), commonly used today. This method is based on measurement of the oscillation frequency of the implant in the bone, induced by a magnetic pulsing stimulus, which is transformed into ISQ values (implant stability quotient) in a range between 1 and 100 [28,29,30]. In vitro studies have shown that the ISQ increases with the stiffness of the bone–implant interface. RFA has proven to be a useful tool for assessing the evolution of implant osseointegration because it allows for clinical measurement of the quality of the bone–implant interface immediately after insertion (primary stability), as well as biological anchorage or secondary stability that occurs in the following weeks [29]. Insertion torque (IT) analysis using a torquemeter is another method that provides information about bone density at the implant placement moment, and to some extent, about implant stability. High values of IT indicate a reduced micromovement in the initial healing period, before osseointegration is achieved in the cortical bone [29,30].

Sennerby and Meredith [31] reported that implant design and surface structure could have an impact on implant stability during initial healing. In a study in dogs, Rompen et al. [32] showed that surface-modified implants maintained implant stability, whilst machined surfaces experienced a decrease in stability during the early healing period. In another study, a machined surface was compared with an oxidized one using an immediate loading protocol, and the authors reported a greater decrease in stability for the machined implants during the first 3 months post-loading [33]. Many researchers have found that implant surface, rough or smooth, has no influence on implant stability [34,35,36,37], although some papers have reported different implant stability with different implant surfaces [38].

Although there are several studies in the literature that compare different surfaces of commercialized implants and their influence on osseointegration and primary stability, we didn’t find any papers in which, on the same commercialized implant, the surface roughness was modified to assess its effect on implant stability.

On the other hand, in relation to implant macro-design, we can distinguish between implants placed at the bone level—at the same level or slightly below the bone crest (bone-level implants)—and those with the active surface of the implant located within the bone, and the coronal portion—a polished titanium ring—on the bone crest (transmucosal); on this supracrestal part is established the soft tissue attachment for tissue-level implants. These transmucosal implants have been shown to represent a valid option in daily practice, particularly when applied in immediate-loading full-arch rehabilitation [39,40]. In this sense, we have not found any primary stability studies comparing different levels of roughness on two different implant designs, tissues, and bone-level dental implants.

The aim of this research was to determine the role of surface roughness and of the type of dental implant (tissue-level or bone-level) on the implant stability, measured via RFA and IT. In addition, the correlation of RFA with IT was analyzed. The null hypothesis was a follows: The surface topography of dental implants, as well as the presence, or lack thereof, of a polished ring at the neck (tissue- or bone-level dental implant), have no influence on their primary stability after insertion into the bone.

This study presents an original study of the primary stability of dental implants using two types of dental implant and, at the same time, with three levels of roughness. The study of different levels of roughness with the same design of dental implants and the comparison between the two types of implant (tissue- and bone-level) are what give this research originality.

## 2. Methods

### 2.1. Dental Implants

**Figure 1:**
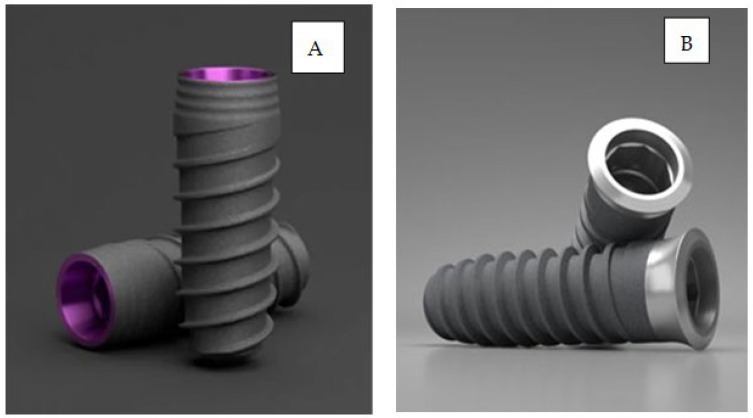
(**A**) Essential dental implant: tissue-level. (**B**) VEGA dental implant: bone-level.

Two hundred and thirty-four implants were employed from Klockner Implant System (SOADCO, Les Escaldes, Escaldes-Engordany, Andorra). Two different implant designs were used: Essential^®^ Cone (internal connection and double-threaded with an atraumatic apex, progressive core, and machined collar (a tissue-level implant)), and VEGA^®^ (internal connection, double-threaded with an atraumatic apex and progressive core designed for placement at the crestal level). The dental implants were made with commercially pure grade 3 titanium. The implants used are shown in Figure 1.

### 2.2. Surface Treatments

Dental implants were prepared with three different surface treatments and divided into six groups (three different surface treatments × two types of dental implant—tissue- and bone-level) with thirty-nine implants for each group. A total of 234 dental implants.

The treatment was realized using alumina particles (120, 300, and 600 µm size) with 0.25 MPa blasting pressure until roughness saturation was achieved. The distance of projection from the gun to the surface was 0.80 m. The different abrasive sizes produced different roughnesses on the surfaces of the dental implants. The sterilization and packing of all implants were carried out at SOADCO, S.L. (Klockner Dental Implants, Escaldes Engordany, Andorra).

The consensus report of implant surfaces and design (working group 4) established three types of surface roughness for oral implants [34], specified by Albrektsson and Wenneberg [35]:

- Minimally rough surfaces: Sa values 0.5–1 µm.
- Moderately rough surfaces: Sa values 1–2 µm.
- Rough surfaces: Sa values > 2 µm.

The implants were distributed among the following groups:

Group A: 39 Essential^®^ Cone minimally rough surface implants.

Group B: 39 VEGA^®^ minimally rough surface implants.

Group C: 39 Essential^®^ Cone rough surface implants.

Group D: 39 VEGA^®^ rough surface implants.

Group E: 39 Essential^®^ Cone moderately rough surface implants.

Group F: 39 VEGA^®^ moderately rough surface implants.

### 2.3. Roughness

White light interferometer microscopy (Wyko NT1100, Veeco, Plainview, NY, USA) was used. Data analysis was performed using Wyko Vision 232^TM^ software (Veeco, Plainview, NY, USA). A Gaussian filter was used to separate waviness and form from the roughness of the surface. Measurements were taken of three different surfaces of each type of surface treatment to characterize the amplitude and spacing roughness parameters, Sa and Pc, respectively. Sa and Pc were calculated by averaging the values of all individual profiles that were evenly distributed along the surface analyzed. A scanning electron microscope (SEM) (JSM 6400, Jeol, Tokyo, Japan) was used to qualitatively analyze the surface topography of the implants.

### 2.4. Wettability

Contact angle analysis was performed with ultrapure distilled water and formamide, and the corresponding data were analyzed using SCA20 (Dataphysics, Filderstadt, Germany). Contact angle measurements were taken using the sessile drop method. Drops were generated using a micrometric syringe and were deposited over discs.

### 2.5. Implant Stability Analysis

**Figure 2:**
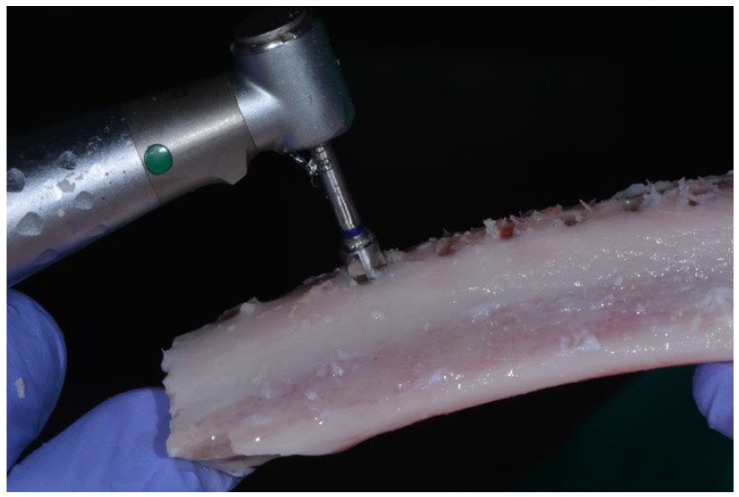
Surgeon placing the implants in fresh bovine bone.

The implants were placed in fresh calf ribs, simulating bone quality type II according to the expertise of the experienced clinician, who performed the insertion of the implants and compared the bone radiographs with the images of the Lekholm and Zarb classification by bone density [41]. All implants used were 10 mm in length and 4 mm in diameter. Figure 2 shows the implantation in bovine bone.

**Figure 3:**
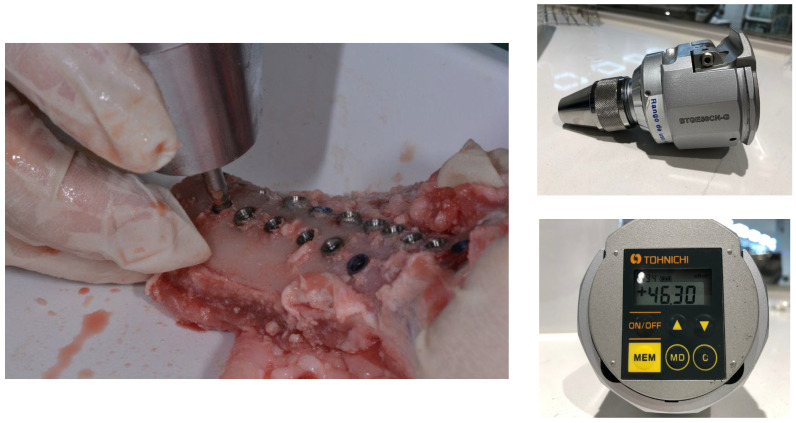
Insertion torque analysis for the determination of primary stability.

The bone site preparations were performed according to the manufacturer’s protocol by an experienced clinician. The protocol began with the use of a sequence of drills, starting with a lanceolated marking bur, continuing with different pilot drills of growing diameter (1.2, 2.8, and 3.5 mm, and finishing with a profile bur. To insert the implant, a low-speed (5 rpm) surgical motor with handpiece was used. After that, a hand torque wrench was used to submerge the implants up the right level. All implants were 4 × 10mm. The osteotomy for each implant was performed under abundant saline solution irrigation with a minimum distance of 4 mm between osteotomies. The rough/smooth interface was placed at the bone crest level for the tissue-level implants (groups A, C, and E), while bone-level implants (groups B, D, and F) were placed at the crestal level. Implant insertion was performed manually, and IT was registered using a calibrated Analog Dynamometer—BTG90CN (Tohnichi, Tokyo, Japan). Figure 3 shows the torquemeter used for the determination of insertion torque.

**Figure 4:**
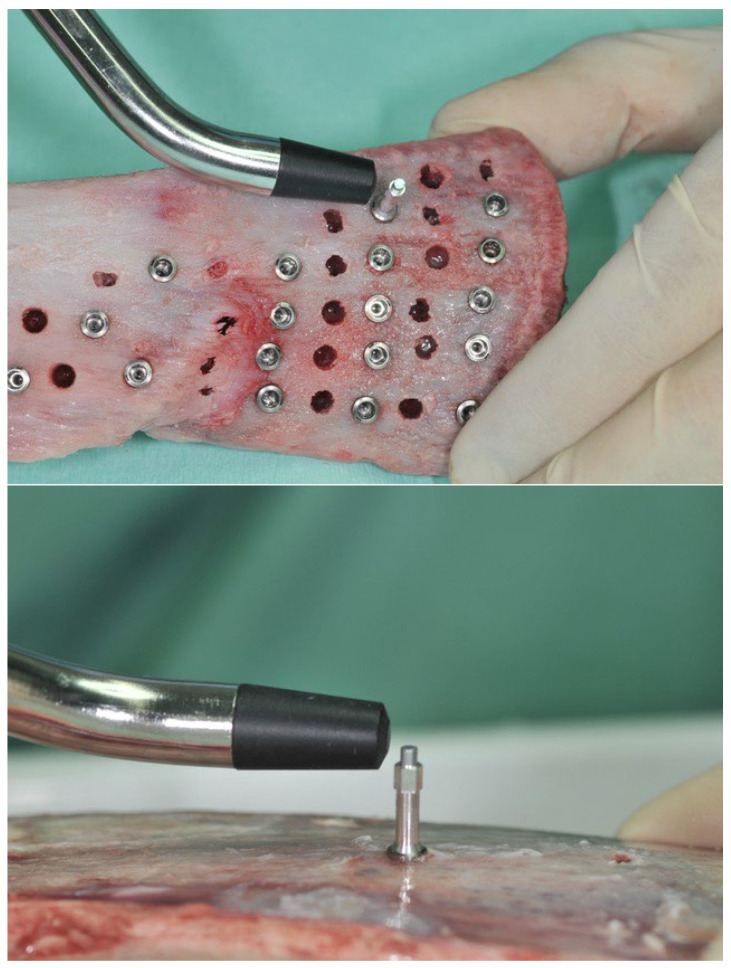
Resonance frequency analysis to determine implant stability quotient.

Once the implants were in place, primary stability was measured by means of resonance frequency analysis (Osstell ISQ^®^), following the manufacturer’s instruction and using the appropriate transducers (SmartPeg) for each implant design (Figure 4). Resonance frequency analysis (RFA) is used to determine primary stability. It is a recent technique but is highly sensitive and non-destructive. This technique determines the stability as a function of the rigidity between the dental implant–bone tissue system by means of a magnetic peg that is connected to the implant. The values range from 1 to 100, and these are the implant stability quotient (ISQ).

The transducers were hand-screwed, at approximately 6–8 N/cm of torque. For each SmartPeg, two measurements were taken at a perpendicular level. Between measurements, the transducer was unscrewed and screwed again. A new SmartPeg was used every 5 implants.

### 2.6. Statistical Analysis

Variables are described in terms of their mean, median, and standard deviation values. Variable normality was checked with the help of the Shapiro–Wilk test. Due to the lack of normality of the variables, two different non-parametric tests were applied. In the case of two categories, the Mann–Whitney–Wilcoxon test was employed [42,43]. In the case of more than two categories, the Kruskal–Wallis test was applied, and in order to find differences among groups, the post hoc Dunn’s test with Holm’s correction was employed [44,45,46].

## 3. Results

**Figure 5:**
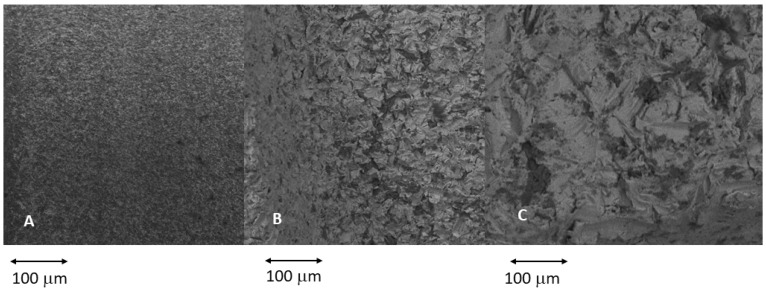
The different topographies studied. (**A**) Minimally rough surfaces: Sa values 0.5–1 µm. (**B**) Moderately rough surfaces: Sa values 1–2 µm. (**C**) Rough surfaces: Sa values > 2 µm.

Figure 5 shows images of the three types of roughness obtained via grit-blasting with different sizes of abrasive alumina particles. As is well known, the projection of larger particles at the same pressure and projection distance generates a higher roughness value. The roughness values obtained for the different surfaces and implants can be seen in Table 1. As expected, the roughness between the two types of implants does not show statistically significant differences.

The contact angles were determined and are shown in Table 2. For the determination of these values, the area index values were corrected. The results are shown in Table 2.

The mean IT of the whole sample is 23.87 ± 11.56 N/cm and the mean ISQ value of the whole sample is 67.12 ± 8.75. The descriptive analysis of the study sample’s rugosity is shown in Table 3. The results obtained show statistically significant differences in the medians among groups for both the IT (H = 96.82.df = 5), with a *p*-value < 2.2 × 10^−16^, and ISQ variables (H = 94.92. df = 5. *p*-value = 2.2 × 10^−16^). The Pearson correlation value of the ISQ and IT variablea is 0.059 (*p*-value = 0.371), which can be considered a low value. All the ISQ values are shown in Supplementary File (Table S1) and the IT values (Table S1) for different roughness and dental implants.

**Figure 6:**
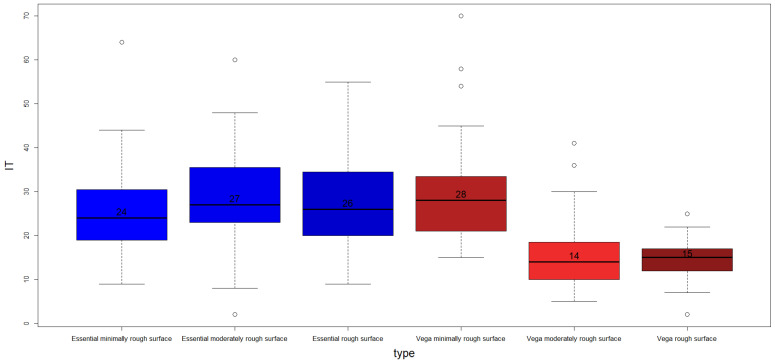
Boxplot of IT by dental implant type. The median values are included.

**Figure 7:**
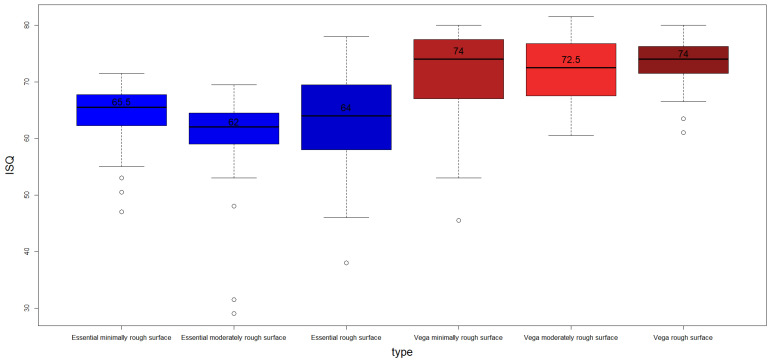
Boxplot of ISQ by dental implant type. It can clearly be observed that the ISQ values are, in general, higher for VEGA dental implants.

Figure 6 shows a box-plot of the IT for each type of implant, and Figure 7 shows a box-plot of the RFA analysis.

Table 4 shows the p-values of the Dunn post hoc test applied to the IT variable with the Holm correction. According to the *p*-values obtained, the different dental implant types can be divided in two categories: Essential minimally rough surface, Essential moderately rough surface, and Essential rough surface versus Vega minimally rough surface, VEGA moderately rough surface, and VEGA rough surface.

The equivalent results for the ISQ variable are presented in Table 5. In this case, the results are similar, also showing differences between VEGA and Essential, but no intra-group differences in either VEGA or Essential.

In order to find out whether there were statistically significant differences among the median values by type of implant (Essential versus VEGA) in the IT and ISQ variables, the Mann–Whitney–Wilcoxon test was applied. Differences among groups were found for both IT (W = 10158.0. *p*-value = 1.50 × 10^−10^) and ISQ (W = 1942.5. *p*-value < 2.2 × 10^−16^). In the case of the IT variable, the median values were 26 for Essential and 27 for VEGA, while for the ISQ variable, the medians were 64 for Essential and 73.5 for VEGA.

Finally, the Kruskal–Wallis test was applied again to find differences among groups by level of roughness. In the case of the IT variable, statistically significant differences among groups were found (H = 18.21. df = 2. *p*-value = 0.0001113). According to the results of the Dunn’s post hoc test, minimally rough dental implants had statistically different median values when compared with moderately rough (*p*-value = 0.00117) and with rough surfaces (*p*-value = 0.000279747). For this variable, the median values obtained were 25 for minimally rough, 20 for moderately rough, and 19 for rough surfaces. In the case of the ISQ variable, no statistically significant differences among groups were found (H = 2.80. df = 2. *p*-value = 0.2462).

## 4. Discussion

The aim of this research was to determine the roles of surface roughness and the type of dental implant (tissue-level or bone-level) on the implant stability, measured via RFA and IT. The first part of the null hypothesis was accepted, i.e., the roughness of the implant surface did not influence the primary stability of the implant. The second part of the hypotheses was rejected, i.e., the presence of a polished ring influenced implant stability.

The experimental results show that the roughness of dental implants does not influence their primary stability values according to the IT and ISQ results. This could be justified by the fact that roughness acts mainly on the secondary stability, i.e., on the biological fixation. The growth of bone tissue on the rough surface favors fixation and causes locking of the implant to the bone as the hard tissue colonizes the entire topography of the dental implant. Gil et al. found that grit-blasting treatment with alumina particles can increase the actual implant surface up to eight times [47]. In other words, the bone will have a larger surface area in contact with the dental implant, and therefore, greater fixation.

Primary fixation is not sensitive to variations in roughness but is more sensitive to the insertion stress exerted by the clinician when placing the dental implant. This is because clinicians drill the bone to a diameter smaller than the implant diameter so that there is compressive stress between the neck of the dental implant and the bone [48]. Clinicians must be careful to ensure that the compressions are not too high, as this can lead to collapse of the blood supply and bone necrosis.

In the same sense, the fact that this research was carried out in an animal model but in vitro also justifies that primary stability depends more on the skill of the clinician than on the surface characteristics, since no scarring process can be initiated. This is reflected in the results of Sul et al. [49], who investigated implant stability using RFA of different topographically or chemically modified implants and four commercially available implants in rabbit bone. The surface properties were characterized in detail, which revealed microstructured, moderately rough implant surfaces varying by 0.7–1.4 mm in Sa. After 6 weeks, all implants showed statistically significantly higher increases in implant stability. The authors report that implant surface properties influenced the RFA measurements of implant stability, but they found no relationship between implant stability and surface roughness. In addition, significant differences in the mean ISQ values were found between the longitudinal and perpendicular measurements of the transducer to the long axis of the tibia at implant placement and after 6 weeks.

Our results show that bone-level dental implants have better primary stability in both the IT and ISQ values. This fact allows us to confirm that there is a good correlation between the stability values obtained by means of the torque teste or by means of RFA. The studies of Ryu et al. already showed some results suggesting the importance of the crestal module in implant designs. In VEGA implants, the neck of the dental implant has a thread design that favors load transfer, and this design also helps the primary fixation of the implant, as has been shown in different studies using finite element analysis and validated by in vivo studies [50]. Similar results were obtained in another study (Menini et al.) [51] in which bone-level implants with triple nanoroughness were also used, in this case extra-short implants (≤6.5 mm), inserted using a one-stage versus two-stage technique in a split-mouth study. The ISQ values were similar in both groups at the time of surgery and had a similar increase at the 12-month follow up appointment.

The configurations of implant collars at the tissue level are polished, which prevents bacteria from adhering to the surface. However, Hangii et al. [52] and Hermann et al. [53] showed that shear forces increase in the crestal region, especially when the collar is placed below the bone crest, causing the resorption of marginal bone tissue. This resorption is due to the lack of mechanical transfer to the bone, and the implant requires the introduction of microthreads as retentive elements in the collar that transfer mechanical loads to the bone. The bone-level implant presents roughness on the entire neck, and this fact favors the transfer of mechanical load to the bone. In addition, microthreads favor this fact, as we have mentioned above. This configuration of the dental implant neck is critical to minimizing marginal bone resorption. This area is very important for the primary fixation of the dental implant, as the transition from an endosteal environment to the oral cavity occurs here [5].

Nowadays, hybrid dental implants are appearing in which the dental implant is composed of two parts: a rough one corresponding to the application zone and a smooth one corresponding to the first threads of the dental implant. The aim of this dental implant is to prevent peri-implantitis due to a smooth surface area, and even if there is a biofilm, the clinician can clean the area via implantoplasty treatment more easily than if the dental implant was totally rough. In our opinion, this implant sacrifices good osseointegration in the area of the implant neck and the possible resorption of marginal bone due to the difficulty of bacterial adhesion and greater ease in cleaning the dental implant in the case of biofilm formation [54,55,56,57,58]. In addition, these implants show differences between compressive residual stresses in the rough area caused by grit-blasting with respect to the smooth area, which facilitates electrochemical corrosion and creates an area in which fatigue crack nucleation is facilitated [59,60].

In this study, we were able to demonstrate that a difference in roughness does not produce significant changes in the primary stability of the dental implant, but the macro-design of the implant does. This fact is important for enabling the clinician to know the characteristics of the different implants, to better choose the type of implant necessary for the patient’s treatment and, more importantly, to decide whether to use implants for immediate loading or early loading.

Regarding the limitations of this study, it was been carried out with only two types of implants, an implant placed at the bone level and another tissue-level-type implant, with a polished ring where the active part would be inside the bone and a ring at the level of soft tissues, with both models marketed by Klockner Implant System (SOADCO, Les Escaldes, Escaldes-Engordany, Andorra). It would have been more ambitious to have other implant designs with different shapes and thread types to analyze how they behave with respect to the measurement of primary stability.

On the other hand, the present study was an in vitro study of an animal model, so it was only possible to measure the immediate bone stability after implant placement (primary stability), but stability could not be measured over time. In an in vivo study, other variables may have an influence that are not present in in vitro studies, even if, as in this case, they are performed on calf ribs. It would be interesting to be able to carry out similar studies on animal models in vivo, in which it would be possible to evaluate whether the same results can be expected and maintained in the long term.

## 5. Conclusions

Different roughness levels, with Sa values from 0.5 to 4 μm, have no influence on the primary stability of dental implants, as determined by IT and ISQ measurements. An increase in roughness produces a light increase in the hydrophilic characteristic of the surface. The macro-design of the dental implant does show an influence, with bone-level dental implants showing higher primary stability compared to tissue-level implants. A good correlation between the primary stability values obtained via IT and ISQ has been demonstrated.

## Figures and Tables

**Table 1:** Values of the different parameters of roughness for the different treatments studied in both types of dental implant.

Samples	Sa (µm)		Sz (µm)		S Area Index	
	Average	Desv.est.	Average	Desv.est.	Average	Desv.est.
A	0.55	0.01	3.47	1.53	1.04	0.01
B	0.54	0.07	16.74	1.11	1.03	0.01
C	3.85	0.18	20.96	1.65	1.74	0.05
D	2.76	0.21	19.65	2.00	1.13	0.07
E	1.60	0.22	13.42	2.54	1.66	0.10
F	1.67	0.19	19.72	3.02	1.88	0.09

**Table 2:** Wettability of the different surfaces.

Samples	A	B	C	D	E	F
Mean	90.88	92.34	76.70	78.45	80.92	82.32
Standard Dev.	5.90	6.00	3.09	2.90	1.85	1.97

**Table 3:** Descriptive statistics of IT and ISQ variables by type of dental implant surface.

Implants	Sa		IT			ISQ		
	Mean	SD	Mean	SD	Median	Mean	SD	Median
A	0.55	0.01	25.85	10.44	24	64.13	5.40	65.50
B	0.54	0.07	29.41	11.84	28	70.77	8.59	74.00
C	3.85	0.18	28.44	11.40	26	63.08	8.12	64.00
D	2.76	0.21	14.69	4.35	15	73.04	4.43	74.00
E	1.60	0.22	29.00	11.01	27	59.68	9.51	62.00
F	1.67	0.19	15.28	7.40	14	72.03	5.73	72.50

**Table 4:** *p*-values of the Dunn post hoc test applied to variable IT with the Holm correction.

	Essential Moderately Rough Surface	Essential Rough Surface	VEGA Minimally Rough Surface	VEGA Moderately Rough Surface	VEGA Rough Surface
Essential minimally rough surface	1	1	1	$5.23\times 10^{-6}$	$2.79\times 10^{-6}$
Essential moderately rough surface		1	0.9106	$2.18\times 10^{-9}$	$9.83\times 10^{-10}$
Essential rough surface			1	$4.44\times 10^{-8}$	$2.05\times 10^{-8}$
VEGA minimally rough surface				$4.17\times 10^{-9}$	$1.93\times 10^{-9}$
VEGA moderately rough surface					1

**Table 5:** *p*-values of the Dunn post hoc test applied to variable ISQ with the Holm correction.

	Essential Moderately Rough Surface	Essential Rough Surface	VEGA Minimally Rough Surface	VEGA Moderately Rough Surface	VEGA Rough Surface
Essential minimally rough surface	0.0462	0.0875	$1.94\times 10^{-4}$	$2.17\times 10^{-5}$	$5.12\times 10^{-7}$
Essential moderately rough surface		0.0536	$1.68\times 10^{-8}$	$1.26\times 10^{-9}$	$7.16\times 10^{-12}$
Essential rough surface			$6.64\times 10^{-5}$	$1.10\times 10^{-5}$	$2.28\times 10^{-7}$
VEGA minimally rough surface				1	0.0978
VEGA moderately rough surface					1

## Data Availability

The data that support the findings of this study are available from the corresponding author upon reasonable request.

## Supplementary Materials

The following are available online at https://www.mdpi.com/article/10.3390/jcm12134190/s1, Table S1: All ISQ and IT data for the different roughness’s and dental implant types studied are shown.
